# Comment on Cimmelli, V.A. Interpretation of Second Law of Thermodynamics in Extended Procedures for the Exploitation of the Entropy Inequality: Korteweg Fluids and Strain-Gradient Elasticity as Examples. *Entropy* 2024, *26*, 293

**DOI:** 10.3390/e27101050

**Published:** 2025-10-10

**Authors:** Samuel Paolucci

**Affiliations:** Aerospace and Mechanical Engineering, University of Notre Dame, Notre Dame, IN 46556, USA; paolucci@nd.edu

**Keywords:** entropy principle, Coleman–Noll procedure, Korteweg fluids

## Abstract

In a recent paper (Entropy 2024, 26, 293), Cimmelli makes use of the constitutive equation of Korteweg fluids to justify the introduction of an extended method to the classical Coleman–Noll procedure. He states that this constitutive equation is incompatible with the second law of thermodynamics and that while several different proposals can be found in the literature to circumvent such a problem, all of them modify the energy balance or entropy flux. Here we note that such statements are not completely true.

## 1. Introduction

Our aim is to clarify and correct the statements made by Cimmelli [1] regarding the incompatibility of the constitutive equation of Korteweg fluids with the second law of thermodynamics (p. 1) and the necessity of modifying the energy equation or entropy flux (p. 4) to overcome this difficulty.

First, we recall that Coleman and Noll [2] developed a systematic procedure for exploiting the entropy inequality. In application of their procedure, most authors (including Coleman and Noll) postulate that the entropy flux and heat flux over the temperature are equal.

Müller [3] shows that while the assumption regarding the entropy flux is true when applied to simple materials, it is not true in general. By taking the entropy flux as an independent constitutive quantity applied to a dipolar material, Müller demonstrates (using the Coleman–Noll procedure) that the generalization of the entropy flux leads to alterations in the theory and leads to complete compatibility with the second law of thermodynamics.

## 2. Discussion

Dunn and Serrin [4] examine capillarity effects in fluids, which are modeled through the presence of density gradients in the elastic part of the Cauchy stress tensor. Such a model was introduced in 1901 by Korteweg [5]. By assuming that the entropy flux is equal to the heat flux over the temperature, they observed that the Korteweg model is not compatible with the second law of thermodynamics unless the material functions in the model vanish identically.

As a possible remedy, Dunn and Serrin [4] propose a modification of the classical energy balance through an additional rate of supply of mechanical energy, the “interstitial working,” supposedly engendered by long-range interactions among the molecules. Thus, Cimmelli [1] is correct in stating that to circumvent the problem (the assumption that entropy flux is equal to heat flux over the temperature), Dunn and Serrin modify the energy equation.

This peculiar modification is also pointed out by Paolucci [6] who also notes that the Korteweg fluid is not a simple material, and thus one needs to consider the entropy flux as an independent constitutive quantity as indicated by Müller [3]. Here we quote the summarizing conclusion of Paolucci’s analysis:

In our work, we use conventional balance equations for mass, linear momentum, energy, and entropy. Using the principle of equipresence, we take constitutive equations to depend on density, temperature, and velocity, and their derivatives up to second order. The Coleman–Noll procedure is applied on the Clausius–Duhem inequality to obtain explicit results for all constitutive quantities up to quadratic nonlinearity. Most importantly, we do not assume that the entropy flux is equal to the heat flux over the temperature.

Paolucci [6], to illustrate his general results, provides the constitutive equations for Korteweg fluids that satisfy the second law of thermodynamics. This is in contrast with the implication of Cimmelli [1]. Furthermore, Paolucci notes that the constitutive equation for the stress tensor proposed by Kortweg [5], including the linear viscous terms, are all present in his Equation (93) that is more general than that of Korteweg and that of the “elastic” material treated by Dunn and Serrin [4] and Cimmelli.

Perhaps Cimmelli [1] misunderstood Paolucci’s (Equation (7) in [6]),

(1) $$\begin{array}{c} k_{i}=\theta \;{\tilde{k}}_{i}=\theta \;h_{i}-q_{i}, \end{array}$$

as modifying the entropy flux. In (1), $h_{i}$ is the entropy flux, $q_{i}$ the heat flux, θ the temperature, $k_{i}$ the temperature-scaled extra entropy flux, and ${\tilde{k}}_{i}$ the proper extra entropy flux. In the above relation, the entropy flux is *not* modified. The relation only serves to simplify the subsequent algebra. Also note that the entropy flux itself $h_{i}$ is obtained directly in the analysis (Equations (58) and (59) in [6]) and that in (1), $h_{i}$, $q_{i}$, and $k_{i}$ are all functions of the complete independent basic fields (Equation (9) in [6]).

## 3. Conclusions

Cimmelli’s [1] statement that Dunn and Serrin [4] modify the energy equation to overcome incompatibility with the second law of thermodynamics is correct. His implication that Paolucci [6] modifies the entropy flux is incorrect. Lastly, Paolucci shows that by considering the entropy flux as an independent constitutive quantity leads to complete compatibility with the second law of thermodynamics using the classical Coleman–Noll procedure consistent with Müller’s [3] analysis and in contrast with Cimmelli’s statement using the Korteweg fluids example. Thus, as long as the entropy flux is considered as an independent constitutive quantity, the classical Coleman–Noll procedure leads to constitutive equations that are fully consistent with the second law of thermodynamics without any extension.
